# Altered Nasal Microbiome in Atrophic Rhinitis: A Novel Theory of Etiopathogenesis and Therapy

**DOI:** 10.3390/microorganisms10112092

**Published:** 2022-10-22

**Authors:** Saurav Sarkar, Fabien Magne, Giriprasad Venugopal, Suvendu Purkait, Naresh V. R. Mutha, Rituparna Maiti, Prity Sharma, Balamurugan Ramadass

**Affiliations:** 1 Department of Otorhinolaryngology and Head Neck Surgery, All India Institute of Medical Sciences, Bhubaneswar 751019, Odisha, India; 2 Microbiology and Mycology Program, ICBM, Faculty of Medicine, University of Chile, Santiago 8320000, Chile; 3 Center of Excellence for Clinical Microbiome Research, All India Institute of Medical Sciences, Bhubaneswar 751019, Odisha, India; 4 Department of Pathology, All India Institute of Medical Sciences, Bhubaneswar 751019, Odisha, India; 5 Department of Pharmacology, All India Institute of Medical Sciences, Bhubaneswar 751019, Odisha, India; 6 Department of Biochemistry, All India Institute of Medical Sciences, Bhubaneswar 751019, Odisha, India

**Keywords:** atrophic rhinitis, nasal microbiome, honey, prebiotics, SCFA

## Abstract

Background: Atrophic rhinitis (AtR) is a chronic nasal condition with polygenic and polybacterial etiology. We investigated the clinical outcomes of honey therapy and the associated nasal microbiome in AtR. Methods: For eight weeks, a nonrandomized control trial using a nasal spray of 10% manuka honey and saline on the right and left sides of the nose was conducted on 19 primary AtR patients. A nasal endoscopy was performed and a mucosal biopsy were taken before and after the intervention. Five of the nineteen patients were selected for microbiome and GPR43 expression studies. Results: We used manuka honey to describe an effective prebiotic treatment for atrophic rhinitis. There were nine males and ten females with an average (±SD) age of 33.8 (±10.7) years. Endoscopic scores and clinical symptoms improved in honey-treated nasal cavities (*p* < 0.003). There was a significant decrease in inflammation, restoration of mucus glands, and increased expression of GPR43 in the nasal cavities with honey therapy. The nasal microbiome composition before and after treatment was documented. Particularly, short chain fatty acid (SCFA) producers were positively enriched after honey therapy and correlated with improved clinical outcomes like nasal crusting, congestion, and discharge. Conclusion: Our approach to treating AtR patients with manuka honey illustrated effective clinical outcomes such as (1) decreased fetid smell, (2) thickening of the mucosa, (3) decreased inflammation with healed mucosal ulcers, (4) increased concentration of the mucosal glands, (5) altered nasal microbiome, and (6) increased expression of SCFA receptors. These changes are consequent to resetting the nasal microbiome due to honey therapy.

## 1. Introduction

Atrophic rhinitis (AtR) is a chronic nasal condition with uncertain etiology, characterized by the formation of thick dry crusts in the roomy nasal cavity, with a fetid smell emanating as a consequence of atrophy of the mucosa and the underlying bone. It affects people with low socioeconomic status residing in conditions of poor hygiene. The disease has a colossal impact on the social life of the patient. Social ostracization is common in patients due to the obnoxious smell emitted from their nose [1,2]. The incidence of primary AtR is common in tropical countries like India, with the highest prevalence seen in the arid areas bordering the great deserts [3,4]. It affects about 1% of the Indian population and is more frequent in young and middle-aged individuals, with a female preponderance.

Over the years, multiple factors have been attributed to the development of this disease. These factors include nutritional deficiency (iron, fat-soluble vitamins, proteins, and phospholipids), autonomic dysfunction leading to excessive vasoconstriction, reflex sympathetic dystrophic syndrome, endocrinal deficiency, and particularly imbalances in estrogen levels, immune dysfunction, and biofilm formation [5,6,7,8]. However, none have been proven to be a direct cause. AtR is essentially diagnosed based on the triad of fetor, greenish crusts, and roomy nasal cavities, typically seen in full-blown cases in later stages. None of the studies established an ideal management protocol for this disease [9,10]. However, various methods suggest controlling nasal dryness by using lubricants and decreasing evaporation from the mucosal surface. Some studies have tried antibiotics with limited success [3]. The variable responses to different treatment regimens are due to the multifactorial nature of this disease [11,12,13]. It does not respond to any particular modality of treatment targeting a specific etiology.

Honey is recognized as a biologic wound-dressing agent with multiple bioactivities that work in concert to expedite the healing process [4,5,6]. Manuka honey used in wound-care products can withstand dilution with substantial amounts of wound exudate and still maintain enough activity to inhibit the growth of bacteria [7]. In this study, we proposed treating AtR by using honey to heal the ulcers and regenerate the mucosa via replenishing the commensal microbiota of the nose.

## 2. Materials and Methods

This study was conducted in a tertiary care hospital, AIIMS Bhubaneswar, Odisha, a state in the eastern region of India, with typical hot and humid conditions throughout the year. This prospective nonrandomized control trial was conducted between 2018 and 2019 and was approved by the Institutional Ethical Committee, AIIMS Bhubaneswar. All research was performed in accordance with the Declaration of Helsinki.

### 2.1. Patient Assessment

Twenty-five patients were screened for the study with a complete medical history, emphasizing the duration of symptoms, drug history, diabetes, douches, multivitamins, head and neck surgery, trauma, and radiation exposure. Thereafter, the patients were clinically investigated with complete blood count, erythrocyte sedimentation rate (ESR), HbA1c, chest X-ray, Mantoux test, venereal disease research laboratory (VDRL) test, nasal endoscopy, nasal mucosal biopsy, computerized tomography (CT) scan of the nose, and paranasal sinuses (PNS) confirming primary AtR.

**Figure 1:**
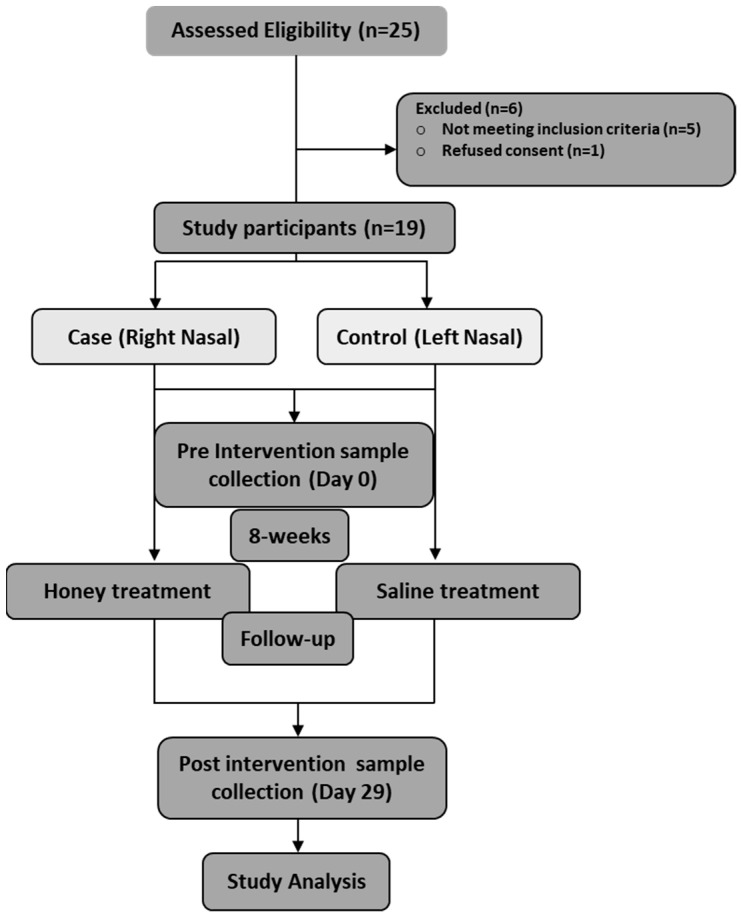
CONSORT: screening and enrolment of study participants.

Nineteen patients were enrolled with the following inclusion criteria (Figure 1, CONSORT): (1) more than 18 years old, (2) having features of AtR in terms of symptoms and clinical examination, and (3) having a similar disease phenotype bilaterally. The exclusion criteria for this study were (1) history of nasal surgery and nasal trauma or irradiation, (2) characteristic systemic or nasal granulomatous disease, (3) history of allergy to honey, (4) known diabetic patients, (5) pregnant and lactating female patients, (6) those lost to follow-up, (7) unwilling to be part of the study, (8) any nasal douches that they might be taking for this condition, and (9) antibiotic use 15 days prior to the intervention.

### 2.2. Clinical Examinations

A nasal endoscopy was performed, crusts were removed, and a score was assigned depending on the clinical features as follows: crusting: gross: 2, minimal: 1, nil: 0; discharge: thick: 2, thin: 1, absent: 0; nasal mucosa: congested: 2, not congested: 1; atrophic turbinate: present: 1, not present: 0; size of nasal cavity: roomy: 2, not roomy: 1. A nasal mucosal biopsy was taken from the left and right sides of the nose on day 1 of the study.

### 2.3. Study Treatment

The right side of the nasal cavity (test) was sprayed with a freshly prepared 10% manuka honey solution, and the left side of the nasal cavity (control) was sprayed with normal saline using separate applicators. The spraying of 10% honey was done twice a week for eight weeks. The participants were instructed not to apply or take medications during this period without informing the principal investigator. At the end of eight weeks, a repeat endoscopic score was assigned to the nasal cavities and biopsied again.

### 2.4. Histological Assessment

Hematoxylin–eosin stain slides were prepared from the paraffin-embedded nasal tissues and were semi-quantitatively assessed for different histological parameters, including granulation tissue and the basement membrane’s thickness, fibrosis, mucus gland, and bacterial colonies. The assessment was done at a single point when the pathologist was blinded to all clinical data. Histologically, all the parameters were graded as absent (0), mild (1), moderate (2), and marked (3). The grades for each parameter were then compared for pre- and post-treatment biopsies. Improvement in the grade by at least two was regarded as an improvement; the cases with a score of 0 were excluded from the further statistical analysis.

### 2.5. Immunohistochemistry

Sections of 3–4 μm in thickness were cut from the paraffin block and mounted on poly-L-lysine coated slides, followed by fixation on a hot plate. The slides were deparaffinized by xylene and rehydrated with a graded alcohol concentration. Antigen retrieval was performed in a 600-watt microwave oven for 30 min using tris ethylenediaminetetraacetic acid (EDTA) buffer (pH 9.0). Next, peroxidase blocking was performed with 3% H_2_O_2_ in methanol for 30 min and incubated with anti-GPR43 antibodies (Invitrogen, Waltham, MA, USA; dilution 1:200) in a humidity chamber for 2 h. Sections were then washed in Tris-buffer, treated with a biotin-labeled secondary antibody for 60 min, and incubated with peroxidase-conjugated streptavidin for 30 min at room temperature. Next, the slides were rinsed with three changes of Tris-HCl buffer followed by diaminobenzidine staining under microscopic control. Again, the slides were rinsed with distilled water, counterstained in hematoxylin for 1 min, and mounted. Skeletal muscle tissue was used as a positive control.

### 2.6. DNA Extraction and 16s rRNA Sequencing

Of the nineteen patients, five patients were selected for microbiome analysis. In total, 20 samples were collected from both sides, including before and after treatment with saline (left-control) and honey (right-test). Paraffin-embedded tissues of 10 µm sections were cut and aliquoted in microcentrifuge tubes and stored at room temperature until extraction. According to the manufacturer’s protocol, DNA was extracted from these samples using QIAGEN’s QIAamp DNA FFPE Tissue Kit (Hilden, Germany). DNA concentration was quantitated using a Nanodrop and qubit fluorimeter and stored at −20 °C until amplification. KAPA HiFi HotStart Ready Mix amplified the hypervariable V3–V4 regions of 16s rRNA genes and a 100 nm final concentration of modified 341F AND 785R primers. The PCR reactions were maintained at 95 °C, 5 min for 25 cycles; 95 °C for 30 s; 55 °C for 45 s; 72 °C for 30 s, and a final extension at 72 °C for 7 min. The amplicons were purified using ampure beads to remove unused primers. Eight additional cycles of PCR were performed using Illumina barcoded adapters to prepare the sequencing libraries. The libraries were quantitated using a Qubit DNA HS quantitation assay (Thermo Fischer Scientific, Grand Island, NY, USA), and sequencing was performed on an Illumina MiSeq (Illumina Inc., San Diego, CA, USA) [8].

### 2.7. Microbiome and Statistical Analysis

The raw reads were checked for quality using FastQC (https://www.bioinformatics.babraham.ac.uk/projects/fastqc/, accessed on 5 July 2020), and reads were trimmed and processed to remove adapter sequences, primers, and low-quality bases using Trimgalore (https://github.com/FelixKrueger/TrimGalore, accessed on 5 July 2020). Preprocessed reads were imported into Mothur V (version 1.35.1; http://www.mothur.org/wiki/Download_mothur, accessed on 5 July 2020), and pairs were aligned to form contigs. The high-quality contigs were checked for identical sequences, and duplicates were merged. The UCHIME algorithm was used to flag contigs with chimeric regions. The filtered contigs were processed and classified into taxonomical units based on the GREENGENES v.13.8-99 database, and contigs were then clustered into OTUs (Operational Taxonomic Units), and relative abundance was estimated.

Raw OTU counts were filtered and normalized by relative log expression. Alpha diversity analysis investigated bacterial species’ diversity between right and left nostrils before and after treatments, categorizing them into four groups. Alpha diversity was evaluated using observed OTUs and Shannon indexes, and then a statistical ANOVA was used to detect whether the index value between the four groups was significantly different. Beta diversity at the species level was evaluated using the PCoA method with the Jensen–Shannon divergence distance method. Alpha diversity estimators and beta-diversity metrics were computed in an online microbiome data analysis platform (Microbiome Analyst) (https://www.microbiomeanalyst.ca/MicrobiomeAnalyst, accessed on 21 February 2021). The relative abundance of bacterial groups was also analyzed using LEfSe (Linear discriminant analysis effect size).

Data analysis and visualization were performed in the R statistical computing environment (Version 4.1.0). The R package *Phyloseq* was used for microbiome analysis (V 1.36.0), and data were visualized using the *ggplot 2* package (V3.3.0). The taxa counts assigned to each sample were tabulated and converted into a phyloseq class. Compositional plots were generated using the transform function. To generate correlation plots, permutational multivariate analysis of variance (PERMANOVA) calculations were performed to detect correlation using the *Vegan* R package. Kruskal–Wallis tests were applied to determine the statistically significant differences in relative abundances of OTU counts. The *p*-value cut-off for the selection of significant differential OTUs was 0.05. A correlation analysis was also performed for significant microbes in the study.

Continuous variables are presented as the mean ± SD and categorical variables as percentages. A comparison of the means of continuous variables (parametric) between two groups was done using a two-sided unpaired *t*-test. Fisher’s exact test was used for categorical variables. The histopathological features of the pre- and post-treatment period are expressed with the median and interquartile ranges. Statistical analyses were performed using statistical software SPSS 22.0 (IBM, New York, NY, USA), considering a significance level of *p* < 0.05.

## 3. Results

Of the nineteen AtR patients recruited, there were ten females and nine males. They were predominantly rural and semi-urban dwellers with a median age of 33.8 ± 10.7 years, hemoglobin percentage of 11.8 ± 1.7 mg, ESR of 18.6 ± 16.2 mm at the end of the first hour, and leucocyte count of 9439 ± 1680 per Cumm. The AtR patients presented with a wide range of symptoms (Table 1). Nasal obstruction was the most common symptom (100%), and epiphora was the least common symptom (5.26%). Epiphora as a presenting symptom has not been reported before in other studies on AtR.

### 3.1. Effects of Intervention

#### 3.1.1. Clinical Endoscopic Findings Improved with Honey Therapy

**Figure 2:**
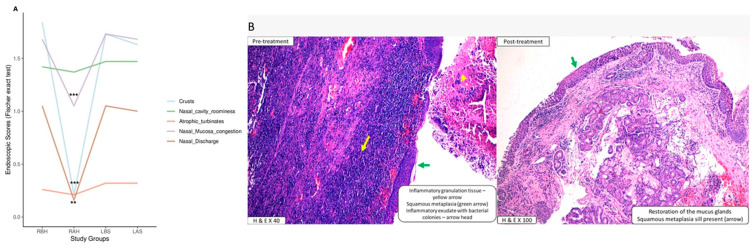
(**A**) Endoscopic scores pre- and post-treatment compared in the right (test) and left (control) sides. (**B**) Restoration of mucosal thickening: the pre- and post-treatment histopathological slide photos showed a decrease in inflammation with a restoration of mucus glands on the right side.

Endoscopic scores were assigned to the right (test) and left (control) sides before and after the intervention. Using Fischer’s exact test, we found that the test side’s nasal crusting, nasal discharge, and nasal mucosal condition improved (*p* = 0.0001). However, the improvement in nasal cavity size and atrophic turbinates was not statistically significant. At baseline, no significant difference in pre-intervention scores was observed between the two sides of the nasal cavity (Table 2, Figure 2A).

#### 3.1.2. Nasal Obstruction, Crust Formation, and Nasal Discharge Improved with Honey Therapy

Patients presented a wide range of symptoms: nasal obstruction, foul smell, impaired smell sensation, crusting, nasal discharge, epistaxis, headache, epiphora, and myiasis in the nasal cavity. To evaluate the effect of honey treatment on AtR symptoms, we performed an interview to assess symptom progression. The symptoms of epiphora and myiasis were excluded as only one patient presented with those. A 2 × 2 contingency table was made, and Fischer’s exact test was applied for each symptom, whether improved or not on the right (test) and left (control) sides. Except for headaches, symptoms were improved in all the cases. The improvement was statistically significant (*p ≤* 0.003) on the test side for nasal obstruction, crust formation, and nasal discharge. No improvement was observed on the control side (Table 3).

Each patient’s cumulative score was noted before and after the intervention and compared between the test and the control: a maximum possible score of 9 for a patient with all nine symptoms and a minimum of 0 for a patient with no symptoms. There was no difference in symptom scores between the test and control before the intervention. However, a statistically significant difference (*p* = 0.0001) in the cumulative symptom score was found between the test and control following the intervention. This indicated an improvement in the test side after the intervention (Table 4).

#### 3.1.3. Honey Therapy Restores Mucosal Glands

A specimen from the nasal cavity was examined histopathologically for the following features: basement membrane thickening, granulation tissue, fibrosis, mucus gland concentration, bacterial colonies, and glandular hypertrophy. Before the intervention, there was no difference in all the features between the test and control. Post-treatment, all the features had improved in the specimens. Granulation tissues, mucus glands, and bacterial colonies were statistically significant (*p* ≤ 0.01) when compared to those in the control side (Table 5).

Emphasis was given to the presence of mucus-secreting glands, as the mucosa’s dryness (due to thinning) and glandular atrophy are the hallmarks of this disease. The median and interquartile range were used to compare the histological features of glandular hypertrophy. The pre-treatment values showed no statistically significant difference between the test and control sides. However, post-treatment values showed significant improvement on the test side (Table 6). Figure 2B shows the decrease in inflammation on the test side, accompanied by a restoration of mucus glands following treatment, compared to that in the pre-treatment side.

#### 3.1.4. Alteration in Nasal Microbiome

We propose that the marked clinical and histological improvement due to honey therapy could be due to the change in the microbiota of the nasal cavity. The nasal microbiome was analyzed to investigate the differences due to honey therapy. In total, the microbiome was evaluated in twenty samples, ten each from the right (test) and left (control) sides, of which five samples each were from before and after the treatment. Each sample group was named as follows: left side before saline treatment (LBS), left side after saline treatment (LAS), right side before honey therapy (RBH), and right side after honey therapy (RAH).

**Figure 3:**
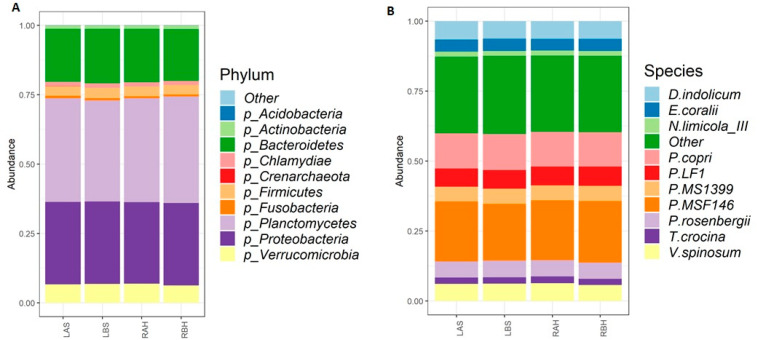
Nasal microbiome composition in AtR: relative abundance of top 10 bacterial taxa at (**A**) phylum-level and (**B**) species-level composition in all four study groups, left side after saline treatment (LAS), left side before saline treatment (LBS), right side before honey treatment (RBH), and right side after honey treatment (RAH). *Desulfobacterium indolicum*; *Enterovibrio coralii*; *Nostocoida limicola* lll; *Prevotella copri*; *Planctomycete* LF1; *Planctomycete* MS1399; *Planctomycete* MSF146; *Photobacterium rosenbergii*; *Tamlana crocina*; *Verrucomicrobium spinosum*.

After quality control and filtering, ~1,266,600 high-quality sequences with an average length of 301 bp were recovered for further analysis, with an average of ~63,330 reads per sample (ranging from 42,908 to 95,343 reads). The rarefaction curves for each sample reached a plateau, indicating sufficient sequencing coverage depth. The 16s rDNA sequencing reads were mapped against the Silva 16s sequence database (v128) and using the RDP classifier identified 506 distinct OTUs. The relative abundance of bacterial taxa at the phylum-level and species-level composition of the nasal microbiome during pre- and post-treatment tests and control are shown in Figure 3. The phyla Planctomycetes (37%) and Proteobacteria (30%) were the most abundant, followed by Bacteroidetes (19%), and the least abundant in either sample before or after treatment was the Vibrionaceae. On core computing, the groups clustered distinctly before and after honey therapy, suggesting an effect of honey on the microbial composition following the intervention. A Venn diagram based on the 80% prevalent species showed that 78 OTUs were shared among all four groups, while 0, 2, 1, and 15 OTUs were found specific to the LBS, LAS, RBH, and RAH, respectively (Figure S1).

**Figure 4:**
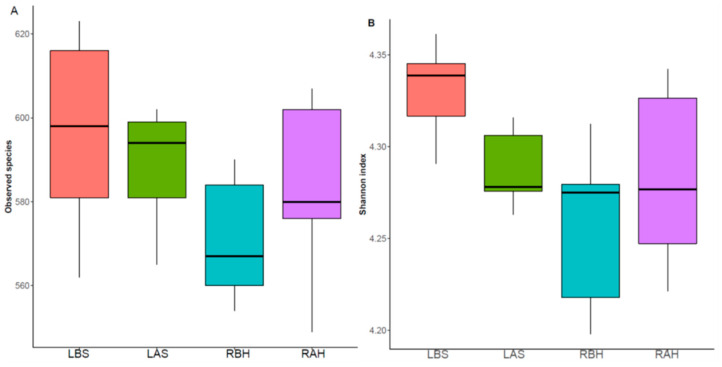
Comparison of alpha diversity of the nasal microbiome before and after honey and saline treatment in atrophic rhinitis patients using an ANOVA test based on (**A**) observed OTUs (*p* = 0.29) and (**B**) Shannon index (*p* = 0.057) metrics.

The alpha diversity of species between the study groups was more evident with the Shannon (evenness and richness) index (*p* = 0.057). At the same time, no significance was observed with the observed (richness) index (*p* = 0.29), as shown in Figure 4. Thus, the alpha diversity was found to be increased after honey therapy when compared to that before honey treatment.

**Figure 5:**
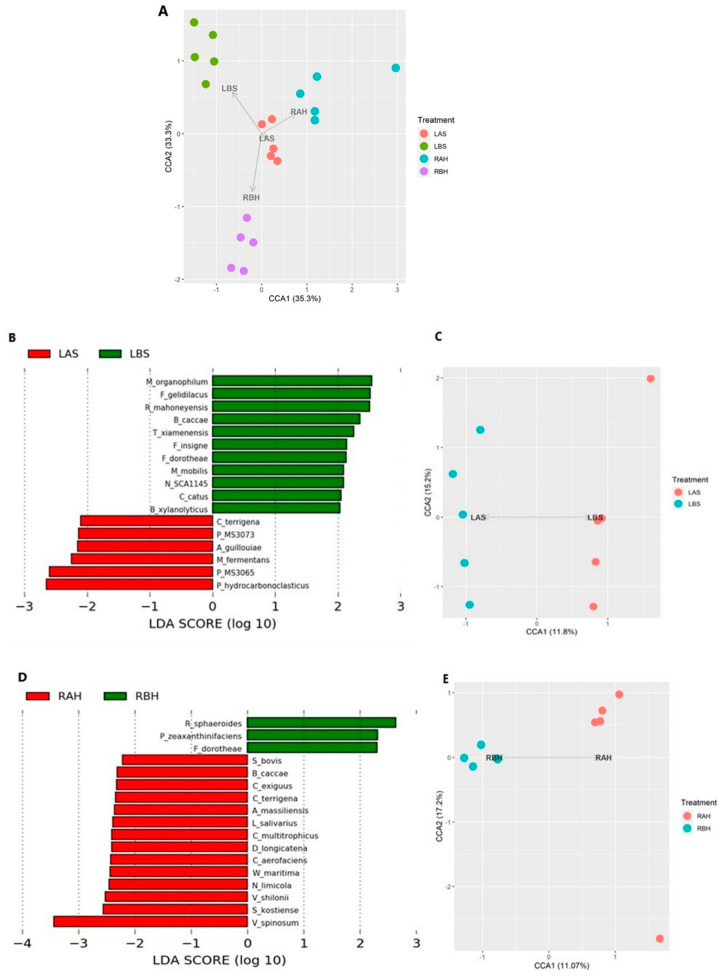
Distinct nasal microbiome: beta-diversity analysis was performed through (**A**) canonical correspondence analysis (CCA) based on Bray–Curtis distances at the species level (PERMANOVA, *p*-value = 0.08). Saline treatment-associated microbiome changes: (**B**) A histogram of log-10-transformed LDA scores was computed for features that showed differential abundance before and after saline treatment. Left side before saline treatment (LBS) and left side after saline treatment (LAS). (**C**) Canonical correspondence analysis (CCA) based on Bray–Curtis distances at the species level (PERMANOVA, *p*-value = 0.2). Improved microbial diversity with honey treatment. (**D**) A histogram of log-10-transformed LDA scores was computed for features that showed differential abundance before and after honey treatment. Right side before honey treatment (RBH) and right side after honey therapy (RAH). (**E**) Canonical correspondence analysis (CCA) based on Bray–Curtis distances at the species level (PERMANOVA, *p*-value = 0.3).

To assess microbial heterogeneity, we evaluated the beta diversity between the study groups (LBS, LAS, RBH, and RAH) using canonical correspondence analysis (CCA) based on Bray–Curtis distances at the species level. All study groups tended to cluster differently on the CCA analysis, and PERMANOVA (*p* = 0.08) did not statistically confirm the trend (Figure 5A). To investigate differentially abundant taxa between study groups, we performed LEfSe analysis to compare the relative abundance of bacterial taxa in LBS, RBH, LAS, and RAH (Figure S2A). Histogram of the linear discriminate analysis (LDA) scores were computed for features. They showed taxa with differential abundance between LBS and RBH (Figure S2B), RBH and LAS (Figure S2C), LBS and RAH (Figure S2D), and LAS and RAH (Figure S2E).

LEfSe analysis between LBS vs. LAS and RBH vs. RAH (Figure 5B,D) identified distinct nasal microbiome patterns for these groups. Meanwhile, the CCA analysis (Figure 5C,E) between LBS vs. LAS and RBH vs. RAH groups clustered separately, displaying distinct nasal microbiome patterns. Fourteen specific bacteria were identified after honey therapy, and only six were identified after saline treatment.

**Figure 6:**
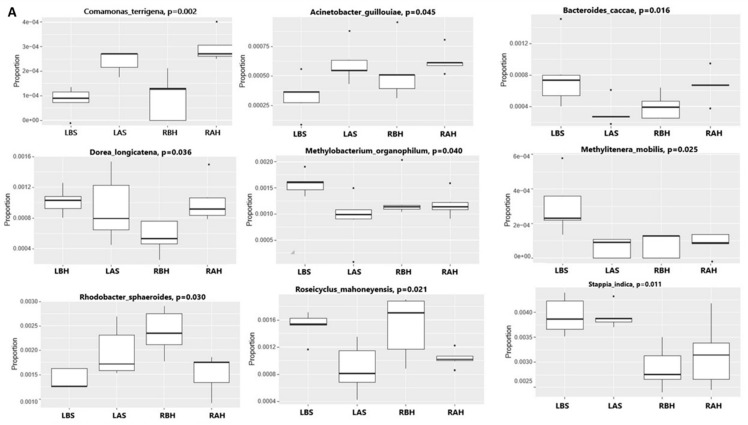

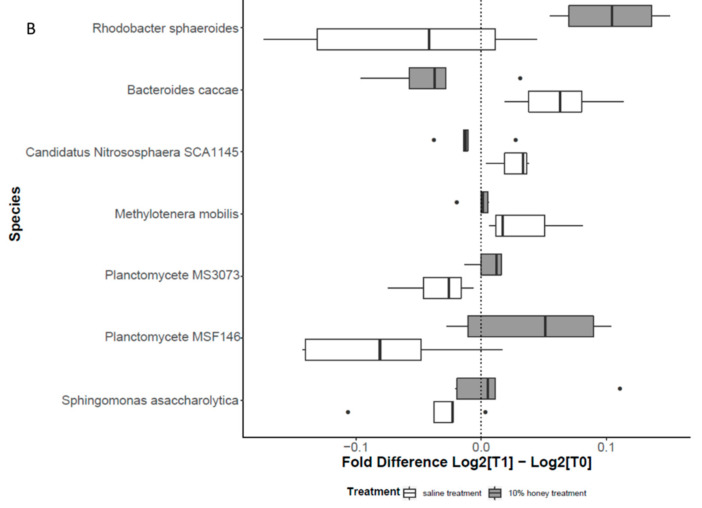
Alteration in nasal microbiome. (**A**) Species-level (excluding species present in <10% of the sample) showing differential abundance between study groups with high significance using the Kruskal–Wallis test; (**B**) Species-level distinction with honey treatment in AtR patients. The fold differences in species before and after honey (**right**) and saline (**left**) treatment.

Differential abundance analysis at all taxonomy levels was performed, and a significant statistical difference was observed between groups. First, the Kruskal–Wallis test was used to test all four groups together (Figure 6A). However, for this strategy, it is crucial to have the same microbiota composition before the treatment on the right and left sides. Second, there is a risk of not detecting some taxa influenced by the treatment, and some taxa might be present that are not stable over time. For these reasons, a second strategy was adopted—the difference between before and after the intervention was calculated (converting the fold difference by log2).

Furthermore, the fold difference between the right (test) and left (control) sides was compared using the Wilcoxon test. The fold difference of some taxa was increased on the test (right) side and decreased on the control (left) side. After honey therapy, we observed increased fold differences in bacteria such as *Rhodobacter spheroides*, *Plancomycete* sp., and *Sphingomonas asaccharolytica* (Figure 6B). In contrast, *Bacteroides caccae*, *Methylotenera mobilis*, *Stapia indica*, *Comamonas terrigena*, *Flexithrix dorotheae*, and *Roseicyclus mahoneyensis* were decreased following honey therapy. Thirdly, we evaluated the associations between the microbial relative abundance after saline (LAS) and honey (RAH) therapy at the species level using multivariate association with PERMANOVA assessing associations between OTUs at the species level and treatment with adjustments for nasal congestion, crust, and nasal discharge (Figure S4). The results indicated that *E. adhaerens*, *P. LF1,* and *T. crocina* were increased markedly in saline treatment, while *P. MSF146, P. copri,* and *V. spinosum* were over-represented in saline treatment.

#### 3.1.5. Increased Expression of SCFA Receptors and SCFA Producers

**Figure 7:**
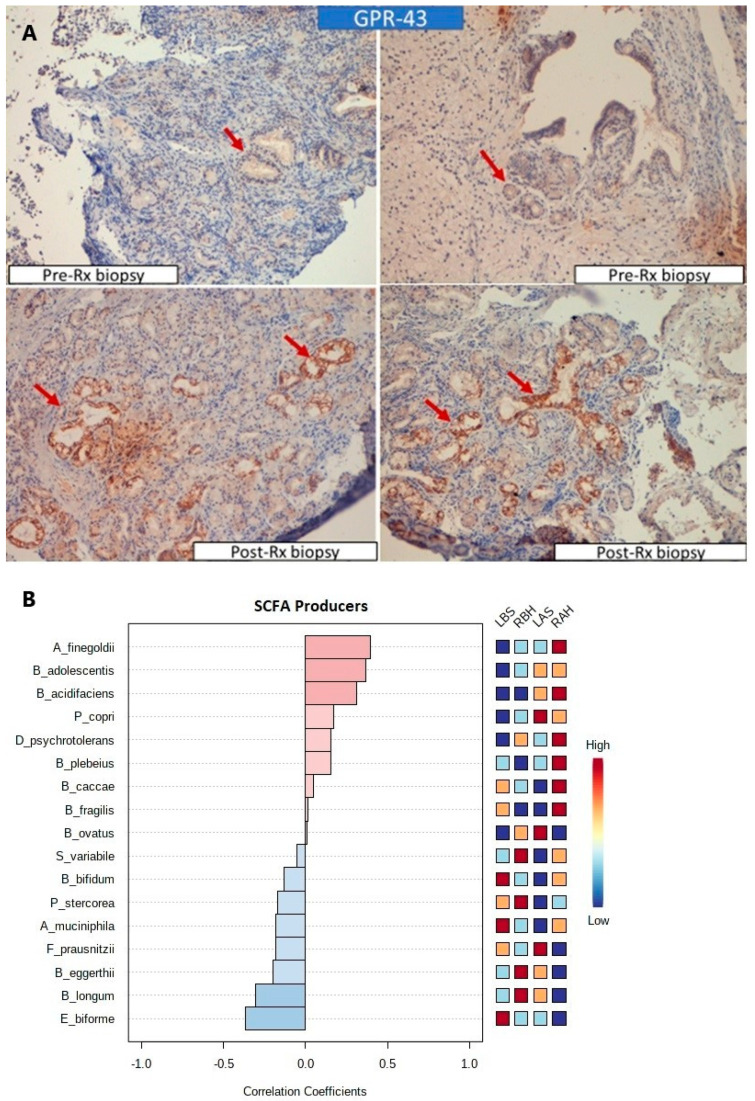
GPR43 overexpression with honey treatment in AtR patients: (**A**) immunohistochemistry pictures showed increased GPR43 receptors in the post-honey-therapy biopsy specimens. (**B**) A mini-heatmap of SCFA producers pattern. Correlation coefficients indicate positive or negative correlations, and colors indicate high (red) or low (blue) relative abundances of the OTUs in the corresponding study groups.

There was a significant difference in microbiome patterns and a remarkable association between clinical improvement and microbiome alteration due to honey therapy. We suspected that the enrichment of the nasal microbiota could be due to the presence of complex carbohydrates in the honey (Figure S3). This alteration in nasal microbiota could increase short-chain fatty acids (SCFAs). A surrogate marker for detecting overproduction of SCFAs is increased SCFA producers [9,10] or the expression of GPR43 receptors [11,12]. GPR43 receptors were over-expressed after honey therapy (Figure 7A). Additionally, SCFA producers like *Alistipes Finegoldii* and *Bacteroides acidifaciens* positively correlated with the RAH group (Figure 7B), while *Akkermansia muciniphila* negatively correlated with the LAS group. Given the improvement with honey therapy, the subjects were prescribed honey on both sides until follow-up. They were followed up clinically after completing the study for eight months, and 18 patients were found to be relieved of the symptoms. One patient did not follow up after the completion of the study.

## 4. Discussion

In this study, manuka honey therapy effectively improved AtR patients. To our knowledge, this is the first study that defines alterations in the nasal microbiome in AtR patients following honey therapy. We found significant improvement in the symptoms’ score of the patients suffering from AtR. We observed a decrease in fetid smell, and the histopathological study showed thickening of the mucosa, decreased inflammation with healed mucosal ulcers, and increased concentration of mucosal glands. The mucosal sparing effect occurred due to honey therapy. In a similar study, honey used in the treatment of AtR showed significant improvement compared to the group in which 25% anhydrous glucose in glycerin was used [13]. However, their study did not specify the type of honey used, and the cause for improvement was not evaluated.

Although attempts to treat this nasal condition started as early as 1971, closure of the nostril (Young’s operation [14]) and endonasal microplasty (intranasal implant insertion) were the surgical management of primary AtR [15]. These options have their demerit of the possibility of decreased quality of life of the patient. Studies using rifampicin, cotrimoxazole, and ciprofloxacin for the disease [3,10,11,16] found rifampicin to be the most effective treatment. In addition, using lubricants like sesame oil to control the signs and symptoms of AtR was also attempted [17,18]. None, however, could establish an ideal or definitive management protocol for the disease. Therefore, even though rifampicin shows promise in treating AtR, in our opinion, its use should be reserved for tuberculosis, especially in developing countries like India, where the prevalence of drug-resistant tuberculosis is 1–3% and is looking to increase [19].

Honey formulations have been used to treat various sinus and nasal inflammations, and accelerated mucosal healing and inflammation and polyp formation reduction have been documented in chronic rhinosinusitis patients [20]. Manuka honey disrupts cellular aggregates, preventing biofilms by a wide range of problematic pathogens [13], including *Streptococcus* and *Staphylococcus* species, *Pseudomonas aeruginosa* [21], *Escherichia coli*, *Proteus mirabilis*, *Enterobacter cloacae*, *Acinetobacter baumannii*, and *Klebsiella pneumonia* [15,16,22].

We hypothesized that manuka honey could have a therapeutic effect on AtR, and pieces of evidence from this study indicate a new theory of etiology of causation of AtR. In AtR, nasal obstruction, fetid smell, impaired smell sensation, crusting, and nasal discharge are caused due to “dysbiosis”. The resident microbes of the nasal cavity in AtR patients may feed on the mucosal (polysaccharides) layer, causing erosion and producing a fetid smell due to the release of sulfur-containing metabolic end-products. This progressive damage may momentarily respond to antibiotics or other therapy that alter microbes or provide lubrication locally. Manuka honey contains a concoction of complex carbohydrates [23,24,25]. In this study, we performed FTIR-ATR spectroscopy on manuka honey, and the spectral peaks matched zymosan A, laminarin, cellulose, cellotriose, and lacto-N-fucopentaose (Figure S4). These carbohydrates promote microbial diversity. The complex carbohydrates of honey provide nutrition for residing microbes, altering the microenvironment of the nasal cavity. The altered microenvironment and constant supply of nutrition (honey) allowed the nasal microbiota to flourish. Correction of “dysbiosis” has a mucosal sparing effect in these patients due to the “colonization resistance” offered by the stabilizing bacteria. An improved mucosa provides the required lubrication, and SCFAs, metabolic end-products produced by the renewed nasal microbiota via utilizing complex carbohydrates, enhance the healing of the ulcers.

To support our theory, we showed an improvement in all the clinical symptoms in all our patients. Mucosal thickening, altered nasal microbiome, and increased GPR43 expression are proofs of a healthier resetting of the nasal microenvironment. GPR43 can be effectively activated by acetate, propionate, and less by butyrate. These SCFAs act as a link between the nasal microbiota and the host response. The epithelial cells may uptake SCFAs both passively and actively; via increased GPR43 expression, they regulate mucin and antimicrobial peptide production, and they can regulate the immune response and growth and differentiation via GPR43 [26]. SCFA producers, particularly *A. finegoldi*, *B. acidifaciens*, *B. Caccae*, and *B. fragilis*, positively correlated with the RAH group and may be responsible for increased GPR43 expression.

The entire population of microorganisms (microbiota) and their genome in an ecologic niche is collectively defined as the microbiome. These ecological niches are sites like the gut, skin, or nasal cavity [27]. Studies suggest that each body’s habitat has its characteristic bacterial community that changes throughout life depending on age. The commensal bacteria in the human nasal cavity suppress opportunistic pathogen colonization by competing for limited nutrients and producing prohibitive metabolites [28,29,30]. Studies suggest that environmental factors modulate the nasal microbiota composition [31,32]. In this study, all AtR patients belonged to low socioeconomic status, were multipurpose laborers, and were exposed to environmental pollutants. Some identified organisms like *C. terrigena*, *A. guillouiae*, and *A. illinoisensis* are known to degrade phenol, isolate from gas-work effluent, and aromatic hydrocarbons from moisturizers, respectively [33,34,35].

Accordingly, we applied bioinformatics and statistical analysis methods comparing groups to validate the suitability of our samples for this investigation. We showed comparable differences in alpha and beta diversity, and statistically significant differentially abundant taxa were shown. LEfSe between honey (RAH) and saline (LAS) treatments provided specific microbial taxa that were statistically discriminative and significant. Fourteen taxa were identified as specific for LAS, and only six were specific for the RAH group. Interestingly, in the LAS group, we identified *B. caccae*, *S. bovis*, *L. salivarius*, and *C. aerofaciens*, which are likely beneficial to the host. We identified several differentially abundant species between two groups after saline and honey treatment (Figure 6A).

An imbalance in the ambient microbial community or dysbiosis could significantly impact human health and is implicated in various diseases and opportunistic infections [26,27,28,36,37]. Nasal dysbiosis has been associated with chronic rhinosinusitis, neurological conditions like Parkinson’s disease, allergic rhinitis, asthma, and otitis media. We observed a significant difference in the nasal microbial profile post-honey-therapy compared to the control side. There is an altered relative abundance of bacterial species *Rhodobacter spheroides*, *Plancomycete* sp., *Sphingomonas asaccharolytica*, *Bacteroides caccae,* *Methylotenera mobilis, Stapia indica, Comamonas terrigena, Flexithrix dorotheae*, and *Roseicyclus mahoneyensis*.

Limitations of the study: A limitation of the study lay in its low sample size and was not a randomized control study. Post-surgical secondary AtR could have been included to increase the ambit of treatment. Formalin-fixed paraffin-embedded tissues were utilized to study the nasal microbiome. However, a snap-frozen tissue would have been an ideal sample. The present study also has its advantages. The case and control being the same person eliminated selection and matching biases. The study concluded with an improvement following the intervention. We tried to search for the reasons for the improvement and, by doing so, developed a new theory of etiology for the disease.

## 5. Conclusions

Manuka honey has a concoction of complex carbohydrates and acts as a prebiotic, promoting a beneficial microbiome of the nasal cavity. A healthy microbiome provides colonization resistance and produces SCFAs, enhancing the healing of the ulcers. As a result, the disease process halts, the mucosa heals, and normalcy returns. Thus, the cure for the disease provided by manuka honey helped us establish the microbiome theory for primary AtR, which can also be extrapolated for secondary AtR, especially that caused after extensive nasal surgery.

## Figures and Tables

**Table 1:** The clinical symptoms of the study patients.

Serial No	Symptom	Number (*n*) of Patients Who Presented with Clinical Symptoms, *n* (%)
1	Nasal obstruction	19 (100%)
2	Foul smell	15 (78.94%)
3	Impaired smell sensation	9 (47.36%)
4	Nasal crust	17 (89.47%)
5	Nasal discharge	12 (63.16%)
6	Maggots	1 (5.26%)
7	Epiphora	1 (5.26%)
8	Epistaxis	3 (15.79%)
9	Headache	5 (26.31%)

**Table 2:** Endoscopic scores pre- and post-treatment compared between the right (test) and left (control) sides.

Endoscopic Feature	Cases				Controls				Cases vs. Control Pre-Treatment Score		Cases vs. Control Post-Treatment Score	
	Pre-Treatment	Post-Treatment	MD 95% CI	*p*-Value	Pre-Treatment	Post-Treatment	MD 95% CI	*p*-Value	MD	*p*-Value	MD	*p*-Value
Crusts	1.84 ± 0.38	0.21 ± 0.42	1.63 SE: 0.13 CI: 1.37–1.89	0.0001	1.73 ± 0.45	1.63 ± 0.49	0.1 SE: 0.15 CI: −0.21–0.41	0.52	0.11 SE: 0.14 CI: −0.16–0.38	0.419	1.5 SE: 0.05 CI: −1.43–1.62	0.0001
Nasal cavity roominess	1.42 ± 0.507	1.37 ± 0.496	0.05 SE: 0.163 CI: −0.28–0.38	0.764	1.47 ± 0.513	1.47 ± 0.513	0 SE: 0.166 CI: −0.39–0.34	1	0.050 SE: 0.165 CI: −0.3–0.39	0.764	0.050 SE −0.053 CI: −0.06–0.16	0.35
Atrophic turbinates	0.26 ± 0.45	0.21 ± 0.42	0.052 SE: 0.14 CI: −0.24–0.34	0.71	0.32 ± 0.48	0.32 ± 0.48	0 SE: 0.16 CI: −0.32–0.32	1	0.05 SE: 0.151 CI: −0.25–0.36	0.73	0.05 SE: 0.05 CI: −0.05–0.15	0.28
Nasal mucosa congestion	1.68 ± 0.48	1.05 ± 0.23	0.630 SE: 0.12 CI: 0.38–0.88	0.0001	1.73 ± 0.45	1.68 ± 0.48	0.05 SE: 0.15 CI: −0.26–0.36	0.743	0.05 SE: 0.15 CI: −0.26–0.36	0.74	0.58 SE: 0.05 CI: −0.48–0.67	0.0001
Nasal discharge	1.05 ± 0.91	0.16 ± 0.37	0.89 SE: 0.23 CI: 0.44–1.35	0.003	1.05 ± 0.91	1 ± 0.88	0.05 SE: 0.291 CI: −0.54–0.64	0.865	0 SE: 0.29 CI: −0.59–0.59	1	0.843 SE: 0.09 CI: 0.67–1.01	0.0001

MD: mean difference, CI: confidence interval, SE: standard error.

**Table 3:** Table showing the degree of improvement in symptoms in the case (right) and control (left) sides of the nasal cavity pre- and post-intervention, and their statistical significance using Fischer’s exact test.

	Improved	Not Improved	*p*-Value
Nasal obstruction (*n* = 19)			
Right	18	1	0.003
Left	9	10	
Foul smell (*n* = 15)			
Right	9	6	0.06
Left	3	12	
Impaired smell sensation (*n* = 9)			
Right	6	3	0.637
Left	4	5	
Crusts (*n* = 17)			
Right	16	1	0.00001
Left	2	15	
Nasal discharge (*n* = 12)			
Right	12	0	0.0013
Left	4	8	
Epistaxis (*n* = 3)			
Right	3	0	0.4
Left	1	2	
Headache (*n* = 5)			
Right	1	4	1
Left	1	4	

**Table 4:** The cumulative symptom scores pre- and post-treatment of the right (test) and left (control) sides of the nose.

	Test/Case Side (Right)				Control Side (Left Side)				Cases vs. Control Pre-Treatment		Cases vs. Control Post-Treatment	
	Pre-Treatment	Post-Treatment	MD 95% CI	*p*-Value	Pre-Treatment	Post-Treatment	MD 95% CI	*p*-Value	MD	*p*-Value	MD	*p*-Value
Cumulative symptom score	4.32 ± 1.92	0.84 ± 1.13	3.47 SE: 0.51 CI: 2.4–4.5	0.0001	4.26 ± 1.88	3.47 ± 2.01	0.79 SE: 0.63 CI: 0.49–2.07	0.22	0.053 SE: 0.66 CI: 1.19–1.3	0.93	2.685 SE: 0.18 CI: 2.3–3.06	0.0001

**Table 5:** Table showing improvement in pathological features in the right (test) and left (control) sides of the nasal cavity pre- and post-intervention, and their statistical significance using Fischer’s exact test.

	Improved	Not Improved	*p*-Value
Basement membrane thinning			
Right (19)	7	12	0.12
Left (19)	2	17	
Granulation tissue			
Right (15)	14	1	0.005
Left (19)	2	17	
Fibrosis			
Right (9)	7	2	0.13
Left (7)	2	5	
Mucus glands			
Right (19)	15	4	0.0001
Left (19)	1	18	
Bacterial colonies			
Right (17)	15	2	0.01
Left (17)	7	10	

**Table 6:** The median and interquartile range of glandular hypertrophy—a pathological feature—pre- and post-treatment compared between the right (test) and left (control) sides.

	Right Nasal Cavity			Left Nasal Cavity			Right Pre-Treatment vs. Left Pre-Treatment	Improvement in Right vs. Improvement in Left
	Pre-Treatment	Post-Treatment	*p*-Value	Pre-Treatment	Post-Treatment	*p*-Value	*p*-Value	*p*-Value
Median	0	15	0.001	1	1	0.32	0.16	0.001
IQR	0	0–2		0–2	0–2			

IQR: Intra quartile range.

## Data Availability

All sequencing data are publicly available on the Sequence Read Archive (SRA) under the study accession number PRJNA682099.

## Supplementary Materials

The following are available online at https://www.mdpi.com/article/10.3390/microorganisms10112092/s1.
