# Supplementary material for: Altered Nasal Microbiome in Atrophic Rhinitis: A Novel Theory of Etiopathogenesis and Therapy
Source: Microorganisms. 2022 Oct 22;10(11):2092. doi: 10.3390/microorganisms10112092 (PMC9694142; doi:10.3390/microorganisms10112092)
Supplement: Supplementary file 1 [file microorganisms-10-02092-s001.zip › microorganisms-1914158-supplementary.pdf]

Supplementary Figures

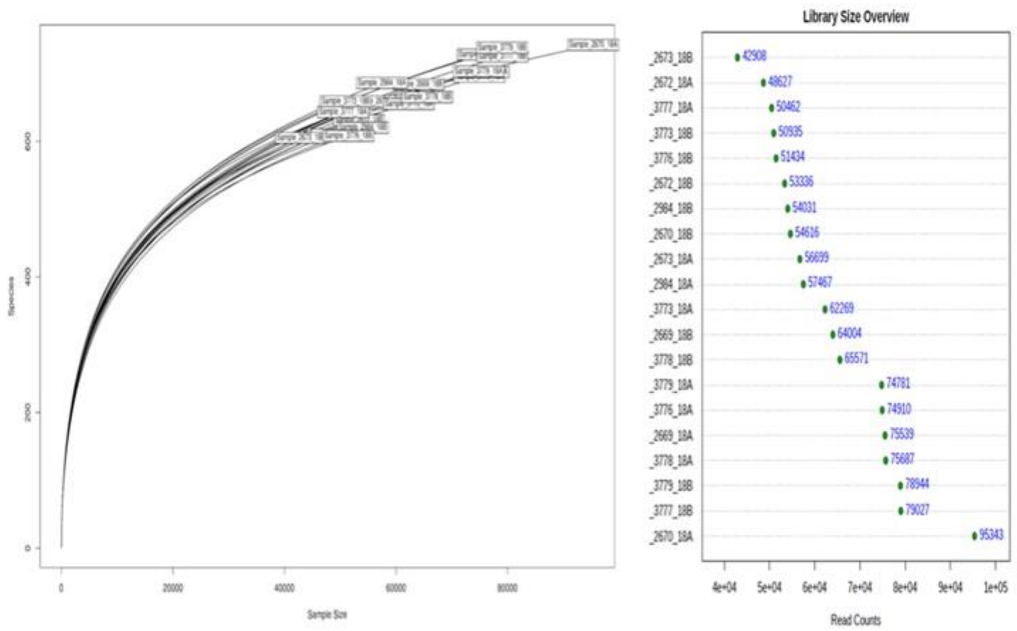

Figure S1: Rarefaction curve shows the measure of diversity that has been captured by a given number of reads per sample, and library overview provides the total number of read counts.

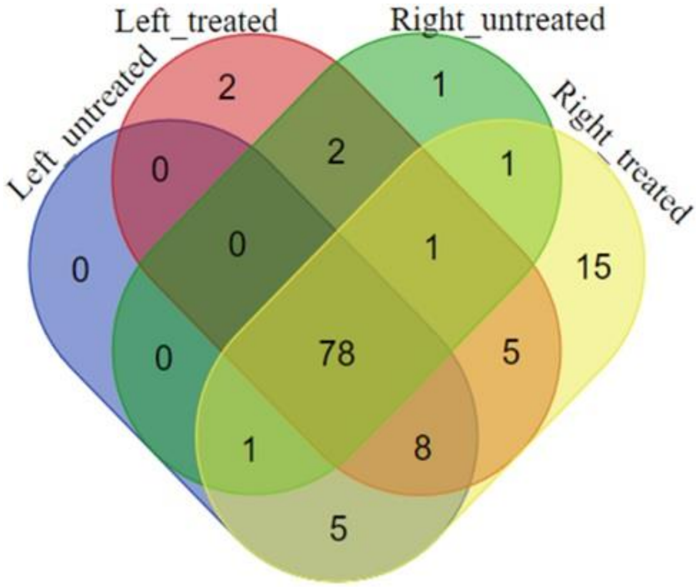

Figure S2: Venn diagram with filtered OTUs with 80% abundance at the species level for each group. We observed that all 4 groups shared 78 OTUs, whereas 0, 2,1, and 15 OTUs were specific to the Left (untreated), Left (Treated), Right (untreated), and Right (treated) groups.

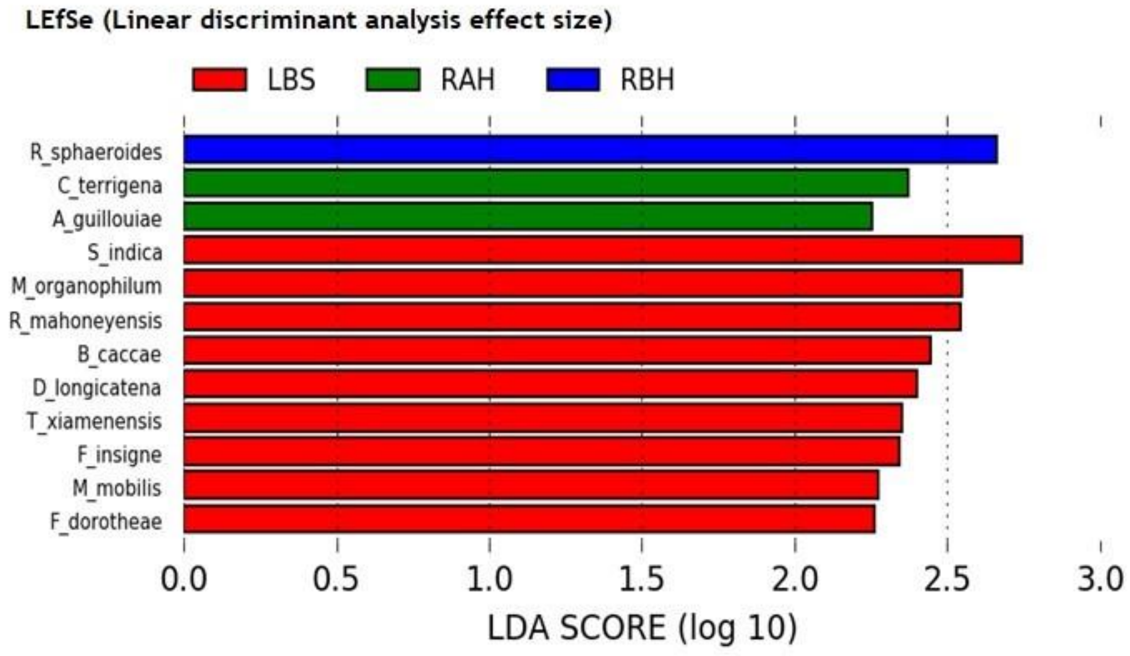

Figure S3A: A histogram of the log 10 transformed LDA scores featuring differential abundance between LBS, LAS, RBH, and RAH.

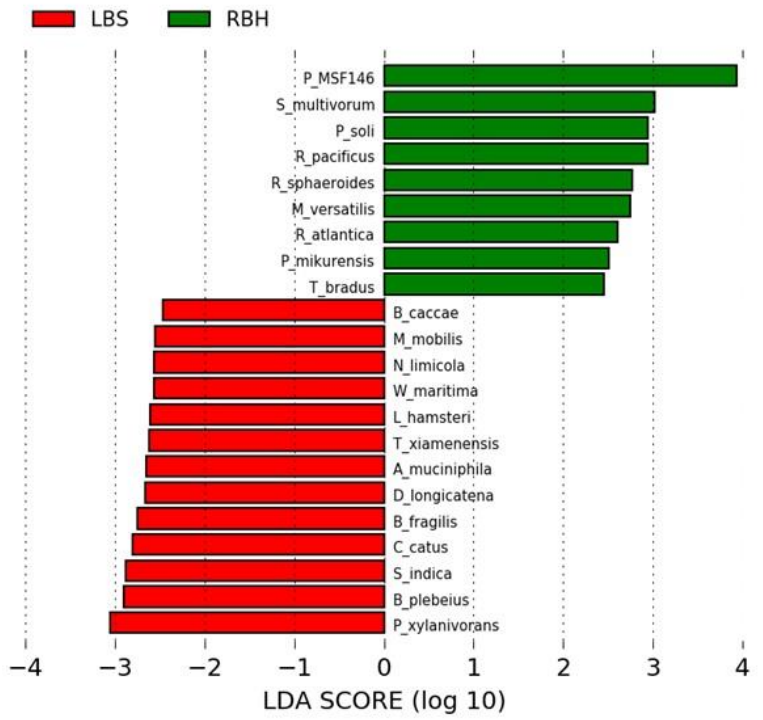

Figure S3B: A histogram of the log 10 transformed LDA scores featuring differential abundance between LBS and RBH.

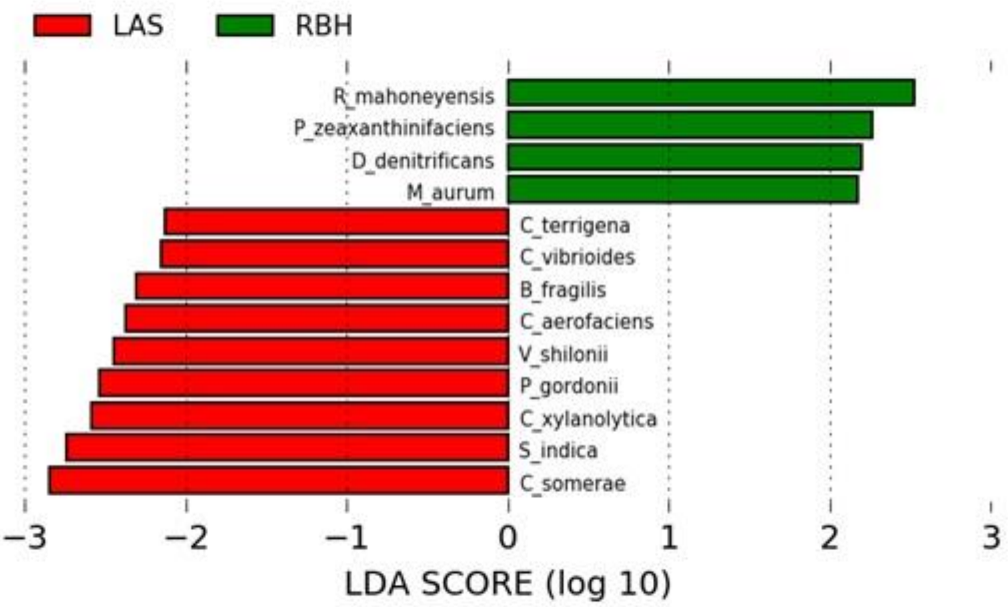

Figure S3C: A histogram of the log 10 transformed LDA scores featuring differential abundance between LAS and RBH.

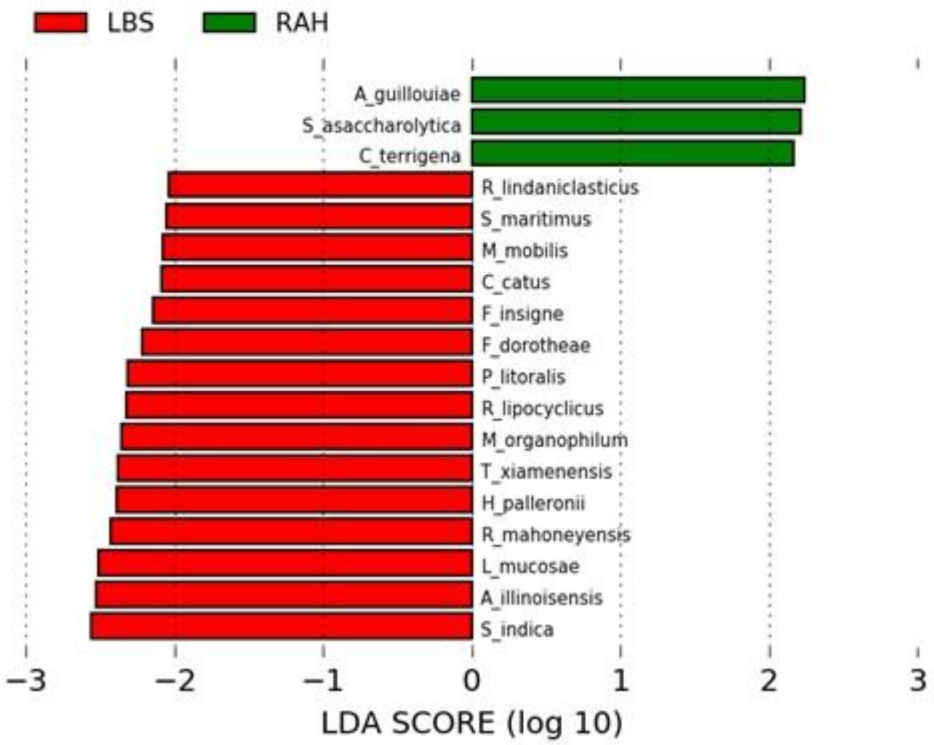

Figure S3D: A histogram of the log 10 transformed LDA scores featuring differential abundance between LBS and RAH.

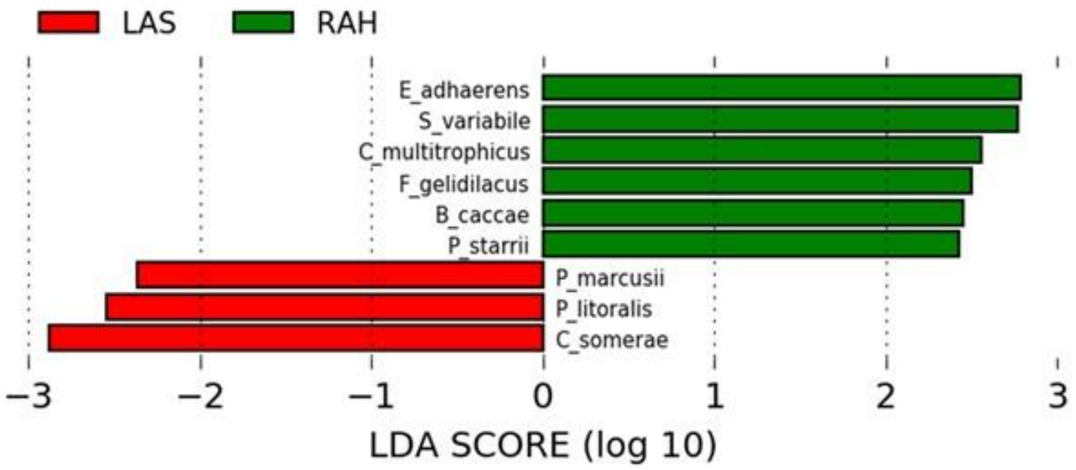

Figure S3E: A histogram of the log 10 transformed LDA scores featuring differential abundance between LAS and RAH.

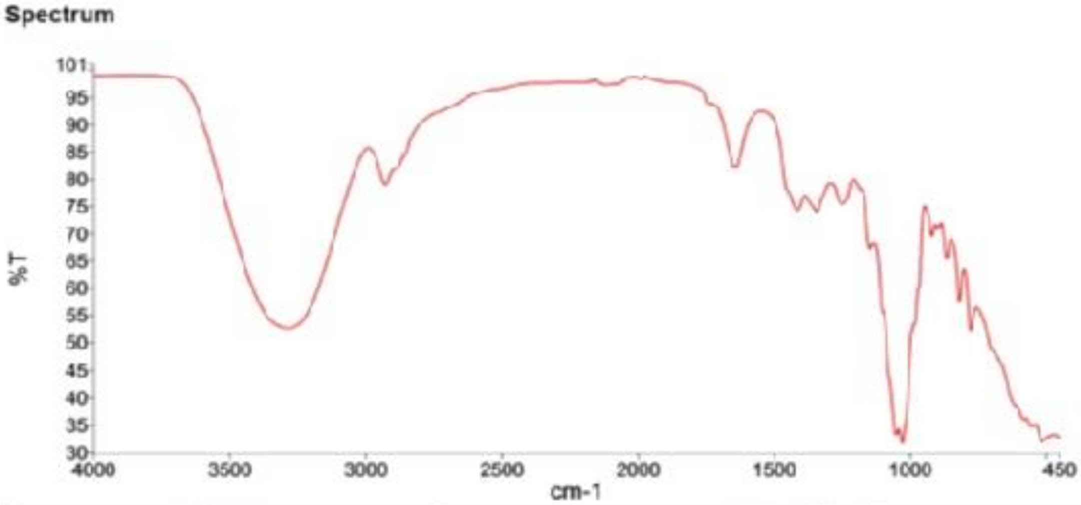

Name	Description
Honey	Sample 067 By OUAT CIF Date Thursday, March 14 2019

Hit Name	Hit Description	Library Name	Library Description	Search Score
PC3523	F97340.SP F97340ZYMOSAN A FROM SACCHAROMYCES CEREVISAE* BIOCHEMIKA	PE_ATR_C		0.845499
PC2062	F61430.SP F61430LAMINARIN FROM LAMINARIA DIGITATA* BIOCHEMIKA	PE_ATR_C		0.844061
AR0102	CELLULOSE	PKEL-S-1		0.815284

Hit Name	Hit Description	Library Name	Library Description	Search Score
PC0898	F22165.SP F22165D (+)-CELLOTRIOSE* BIOCHEMIKA C18H32O16	PE_ATR_C		0.808826
PC0178	F03494.SP F03494L ACTO-N-FUCOPEN TAOSE I* BIOCHEMIKA C32H55NO25	PE_ATR_C		0.801566
PC0177	F03493.SP F03493L ACTO-N-DIFUCOHE XAOSE II* BIOCHEMIKA C38H65NO29	PE_ATR_C		0.798629

Figure S4: ATR Spectral pattern of manuka honey and the best match corresponding to the ATR spectra.

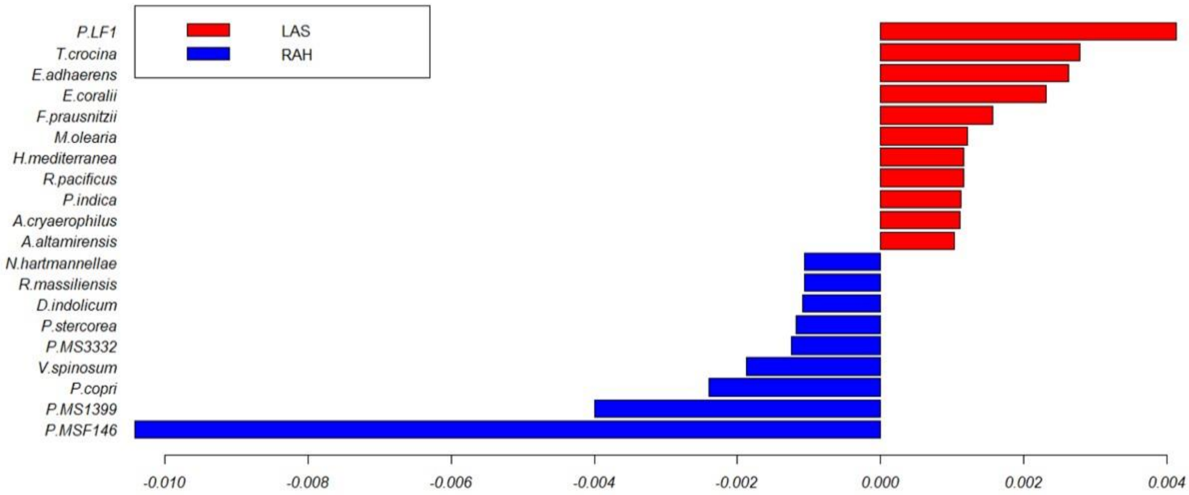

Figure S5: **Distinct Nasal Microbiome:** Bar plot of r-coefficients assessing associations between OTUs at Species-level and treatment with adjustments for nasal congestion, Crust, and Nasal discharge. Positive (red bar) and negative (blue bar) are coefficient values representing taxa enriched in honey and saline-treated groups.
